# Insertion-and-Deletion Mutations between the Genomes of SARS-CoV, SARS-CoV-2, and Bat Coronavirus RaTG13

**DOI:** 10.1128/spectrum.00716-22

**Published:** 2022-06-06

**Authors:** Tetsuya Akaishi

**Affiliations:** a Division of General Medicine, Tohoku University, Sendai, Japan; b Department of Education and Support for Regional Medicine, Tohoku University, Sendai, Japan; c COVID-19 Screening Test Center, Tohoku University, Sendai, Japan; University of Siena

**Keywords:** bat coronavirus RaTG13, coronavirus disease 2019 (COVID-19), mutation, insertion-and-deletion mutation, severe acute respiratory syndrome coronavirus (SARS-CoV), severe acute respiratory syndrome coronavirus 2 (SARS-CoV-2)

## Abstract

The evolutional process of severe acute respiratory syndrome coronavirus 2 (SARS-CoV-2) development remains inconclusive. This study compared the genome sequences of severe acute respiratory syndrome coronavirus (SARS-CoV), bat coronavirus RaTG13, and SARS-CoV-2. In total, the genomes of SARS-CoV-2 and RaTG13 were 77.9% and 77.7% identical to the genome of SARS-CoV, respectively. A total of 3.6% (1,068 bases) of the SARS-CoV-2 genome was derived from insertion and/or deletion (indel) mutations, and 18.6% (5,548 bases) was from point mutations from the genome of SARS-CoV. At least 35 indel sites were confirmed in the genome of SARS-CoV-2, in which 17 were with ≥10 consecutive bases long. Ten of these relatively long indels were located in the spike (S) gene, five in nonstructural protein 3 (Nsp3) gene of open reading frame (ORF) 1a, and one in ORF8 and noncoding region. Seventeen (48.6%) of the 35 indels were based on insertion-and-deletion mutations with exchanged gene sequences of 7–325 consecutive bases. Almost the complete ORF8 gene was replaced by a single 325 consecutive base-long indel. The distribution of these indels was roughly in accordance with the distribution of the rate of point mutation rate around the indels. The genome sequence of SARS-CoV-2 was 96.0% identical to that of RaTG13. There was no long insertion-and-deletion mutation between the genomes of RaTG13 and SARS-CoV-2. The findings of the uneven distribution of multiple indels and the presence of multiple long insertion-and-deletion mutations with exchanged consecutive base sequences in the viral genome may provide insights into SARS-CoV-2 development.

**IMPORTANCE** The developmental mechanism of severe acute respiratory syndrome coronavirus 2 (SARS-CoV-2) remains inconclusive. This study compared the base sequence one-by-one between severe acute respiratory syndrome coronavirus (SARS-CoV) or bat coronavirus RaTG13 and SARS-CoV-2. The genomes of SARS-CoV-2 and RaTG13 were 77.9% and 77.7% identical to the genome of SARS-CoV, respectively. Seventeen of the 35 sites with insertion and/or deletion mutations between SARS-CoV-2 and SARS-CoV were based on insertion-and-deletion mutations with the replacement of 7–325 consecutive bases. Most of these long insertion-and-deletion sites were concentrated in the nonstructural protein 3 (Nsp3) gene of open reading frame (ORF) 1a, S1 domain of the spike protein, and ORF8 genes. Such long insertion-and-deletion mutations were not observed between the genomes of RaTG13 and SARS-CoV-2. The presence of multiple long insertion-and-deletion mutations in the genome of SARS-CoV-2 and their uneven distributions may provide further insights into the development of the virus.

## INTRODUCTION

Since the emergence of the coronavirus disease 2019 (COVID-19) caused by severe acute respiratory syndrome coronavirus 2 (SARS-CoV-2), the evolutionary process of the virus has been rigorously discussed (1–5). Elucidating the mechanism of its emergence may be important for not only effectively dealing with the current pandemic with intermittent appearance of consequential variant strains but also preventing the occurrence of future outbreaks of different emerging infectious diseases in the future. Currently, COVID-19 is considered a zoonotic disease, and its progenitor is considered to have emerged and maintained in bats as natural reservoir hosts (6), as the same with severe acute respiratory syndrome coronavirus (SARS-CoV) in 2002–2003 (7). This hypothesis is supported by the more than 95% similarity between the genome sequences of SARS-CoV-2 and bat coronavirus RaTG13, extracted from Rhinolophus affinis (8). The supposed progenitors of SARS-CoV-2 were also seen in Malayan pangolins, which also could have played some roles in maintaining the progenitor viruses in natural environments (9). Sometime between 2013 and 2019, the progenitor virus gained a functional polybasic furin cleavage site at the boundary region between S1 and S2 domains of the spike (S) gene through the insertion of four amino acid residues of “-PRRA-” (10). This produced the furin cleavage motif with “-RRAR-” at the S1/S2 boundary area, after which the furin or related proteases are believed to efficiently cleave the protein after entering the host cells (11). This insertion at the S1/S2 boundary has not been confirmed in the potential progenitor viruses including SARS-CoV, RaTG13, or pangolin coronaviruses (such as GD/P1L and GD/P2S), which is a strong rationale for the critical role of the acquired polybasic cleavage site in adapting to sustained human-to-human transmission of SARS-CoV-2 (12, 13). Furthermore, another plausible hypothesis for the emergence of SARS-CoV-2 is a highly variable genome sequence in the receptor-binding domain (RBD) of the S1 gene (14, 15), which could have contributed to the increased binding of S protein to the human receptor angiotensin-converting enzyme 2 (ACE2) and the enhanced immune evasion of human immunity to SARS-CoV-2 (16–18). Although the acquisition of these additional characteristics of the virus has been proposed as a promising scenario for SARS-CoV-2 development, the types and incidence of mutations across the whole viral genome behind the evolution of the virus have not been fully evaluated. This study aimed to gain a deep insight into the evolutionary process of lineage B SARS betacoronavirus by comparing the reference genome sequences and mutations occurring in both the coding and noncoding regions of SARS-CoV, RaTG13, and SARS-CoV-2.

## RESULTS

### Overall mutations between the genome of SARS-CoV-2 and SARS-CoV.

Details of the insertion and/or deletion (indel) mutations and point mutations (substitutions) in the whole genome of SARS-CoV-2, compared to the genome sequence of SARS-CoV, are summarized in Table 1. In the 29,903 bases of the genomes of SARS-CoV-2, 5,548 (18.6%) bases were mutated based on point mutations and 1,068 (3.6%) bases were mutated based on indels. In total, the genome sequence of SARS-CoV-2 was 77.9% (23,287 of the 29,903 bases) identical to that of SARS-CoV. The rate of point mutation in the coding regions was relatively equal between the listed genes, but it was significantly higher than that in the noncoding regions (*P* < 0.0001, chi-square test). In contrast to the relatively even distribution of point substitution across the genome sequence, indels were disproportionally distributed across the coding regions. The rate of indels in each gene was the lowest with 0.0% in the envelope (E), open reading frame (ORF) 6, ORF7, nucleocapsid (N), and ORF10 genes, whereas it was the highest with 90.7% in ORF8 gene. At least 35 indel sites were confirmed in the genome of SARS-CoV-2, in which 17 indels were with ≥10 consecutive bases long. Ten of these relatively long indels were located in the S gene, five in nonstructural protein (Nsp) 3 gene of ORF1a, and one in ORF8 and noncoding region. Seventeen (48.6%) of the 35 indels were based on insertion-and-deletion mutations (that is, insertion and deletion mutations were simultaneously occurred at exactly the same position) with exchanged gene sequences of 7–325 consecutive bases. Furthermore, point mutation patterns between the whole genomes of SARS-CoV and SARS-CoV-2 were evaluated (Table 2). C > T (18.7%) substitution was more frequent than other types of substitution, while G > C (2.2%) and C > G (2.2%) substitution frequencies were lower than those of the others. The base compositions within the indel mutations in the genomes of SARS-CoV and SARS-CoV-2 are summarized at the bottom of Table 2. The base composition within the indels was largely preserved between the two viruses.

**TABLE 1:** Composition of the indel mutations and point substitutions of the genome of SARS-CoV-2, compared to that of SARS-CoV^*a*^

Gene regions	Total no. of bases (Wuhan-Hu-1)	Base counts with indels	Rate of indel mutations	No. of bases after excluding indels	Base counts with point substitutions	Point substitution rate^*b*^
ORF1ab	21,290	366	0.0172	20,924	4,120	0.1970
S gene	3,822	315	0.0824	3,507	809	0.2307
ORF3	828	8	0.0097	820	196	0.2390
E gene	228	0	0	228	12	0.0526
M gene	669	3	0.0045	666	98	0.1471
ORF6	186	0	0	186	43	0.2312
ORF7	494	0	0	494	83	0.1680
ORF8	366	332	0.9071	34	4	0.1176
N gene	1,260	0	0	1,260	142	0.1127
ORF10	117	0	0	117	8	0.0684
All noncoding regions	643	44	0.0684	599	33	0.0551
Total	29,903	1,068	0.0357	28,835	5,548	0.1924

a The shown numbers of bases are for the genome sequence of severe acute respiratory syndrome coronavirus 2 (SARS-CoV-2) with the whole genome sequence of 29,903 bases. Different from the point substitutions in the coding regions, indels were uneven distributed across the whole genome sequences. SARS-CoV, severe acute respiratory syndrome coronavirus; ORF, open reading frame.

b Denominator of the rate of point substitution was the number of bases after excluding the insertion and/or deletion (indel) mutations in each of the gene regions.

**TABLE 2:** Mutation profiles between the genome sequence of SARS-CoV and SARS-CoV-2^*a*^

Coronavirus species	Adenine (A)	Thymine (T)	Guanine (G)	Cytosine (C)	Total
SARS-CoV (2002–2003) genome base composition					
Base count	8,476 (28.5%)	9,135 (30.7%)	6,186 (20.8%)	5,939 (20.0%)	29,736 (100.0%)
SARS-CoV-2 (2019) genome base composition					
Base count	8,954 (29.9%)	9,594 (32.1%)	5,863 (19.6%)	5,492 (18.4%)	29,903 (100.0%)
Single base substitution (vertical > horizontal bases) between SARS-CoV and SARS-CoV-2, base count (%)					
	(SARS-CoV-2)				
A (SARS-CoV)		617 (11.1%)	473 (8.5%)	251 (4.5%)	1,341 (24.2%)
T (SARS-CoV)	649 (11.7%)		195 (3.5%)	710 (12.8%)	1,554 (28.0%)
G (SARS-CoV)	722 (13.0%)	289 (5.2%)		124 (2.2%)	1,135 (20.5%)
C (SARS-CoV)	359 (6.5%)	1,039 (18.7%)	120 (2.2%)		1,518 (27.4%)
Total	1,730 (31.2%)	1,945 (35.1%)	788 (14.2%)	1,085 (19.6%)	5,548 (100.0%)
Base composition within the insertion and/or deletion mutation sites, base count (%)					
SARS-CoV	259 (28.9%)	261 (29.1%)	199 (22.2%)	177 (19.8%)	896 (100.0%)
SARS-CoV-2	349 (32.7%)	331 (31.0%)	185 (17.3%)	203 (19.0%)	1,068 (100.0%)

a The compositions of the 5,548 point mutations between the whole genome sequences of SARS-CoV in 2002–2003 and the subsequent SARS-CoV-2 in 2019 are shown. C > T (18.7%) substitution was more frequent than other types of substitution, whereas G > C (2.2%) and C > G (2.2%) substitutions were less frequent than others. SARS-CoV, severe acute respiratory syndrome coronavirus; SARS-CoV-2, severe acute respiratory syndrome coronavirus 2.

### Overall mutations between the genome of RaTG13 and SARS-CoV.

Next, the whole genome sequences between bat coronavirus RaTG13 and SARS-CoV were compared. The genome sequence of RaTG13 was 77.7% (23,204 bases of the 29,855 bases) identical to the genome of SARS-CoV. In the 6,651 bases of RaTG13 with mutations from SARS-CoV, 1,019 bases (15.3%) were derived from indels and 5,632 (84.7%) were derived from point mutations. Most of the indels between the genomes of SARS-CoV-2 and SARS-CoV, as described in the previous section, were also confirmed between the genomes of RaTG13 and SARS-CoV.

### Overall mutations between the genome of SARS-CoV-2 and RaTG13.

Next, the whole genome sequences between SARS-CoV-2 and RaTG13 were compared. The genome sequence of SARS-CoV-2 was 96.0% (28,720 of the 29,903 bases) identical to that of RaTG13. The mutated 1,183 bases were comprised of 1157 (97.8%) with point mutations and 26 (2.2%) with indels. Importantly, long insertion-and-deletion mutations, which were present between the genomes of SARS-CoV-2 and SARS-CoV, were absent between SARS-CoV-2 and RaTG13 genomes.

### Mutations in SARS-CoV-2 spike gene.

**FIG 1:**
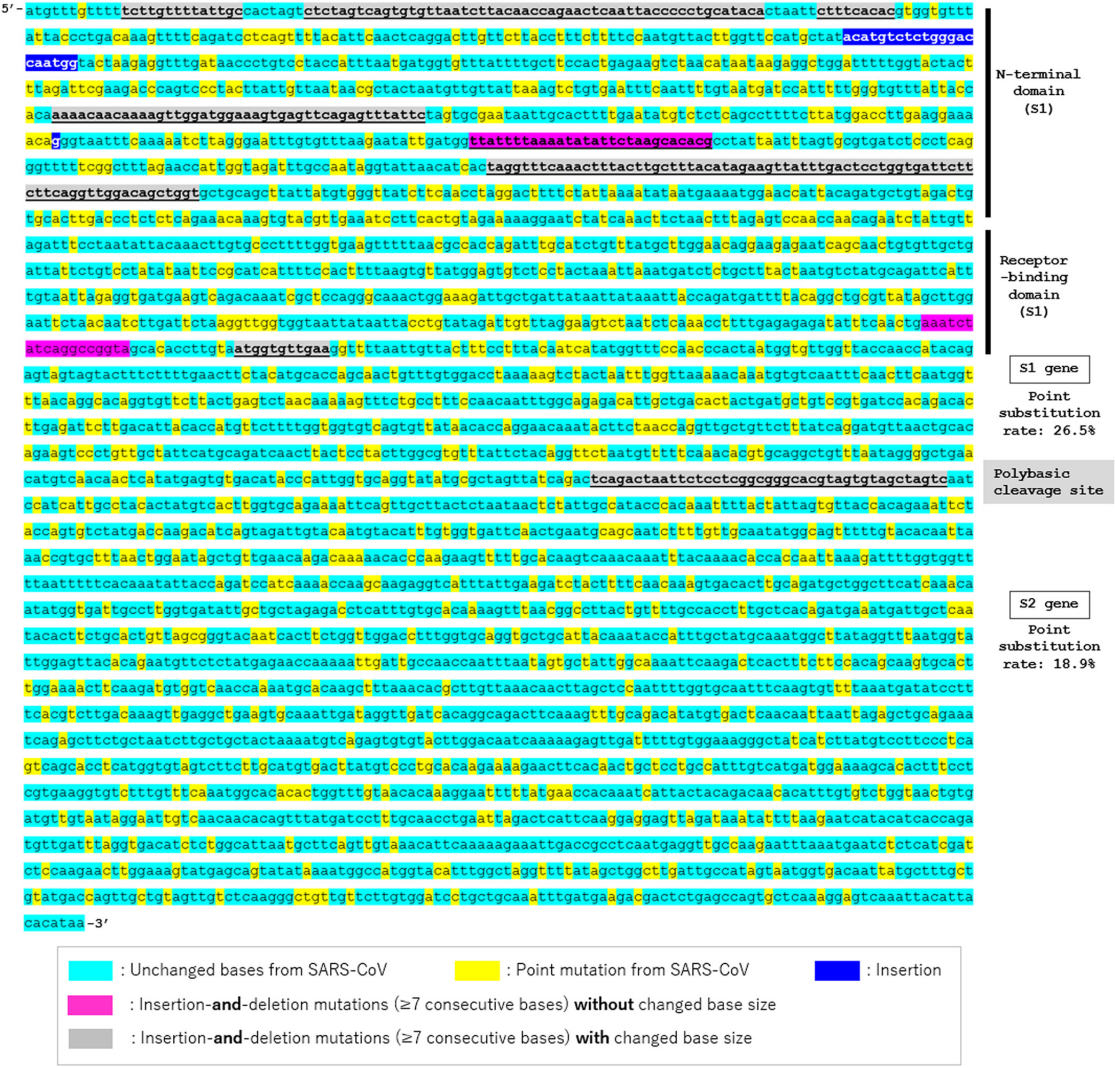
Spike gene sequence of severe acute respiratory syndrome coronavirus 2 (SARS-CoV-2) and mutation types in severe acute respiratory syndrome coronavirus (SARS-CoV). A comparison of S gene sequence between SARS-CoV and SARS-CoV-2 revealed that at least seven 14–245 base-long insertion-and-deletion mutations were concentrated in the N-terminal domain (NTD) of the S1 gene. The shown point substitution rates (26.5% for S1 and 18.9% for S2) are for the sequences excluding the insertion and/or deletion sites. If these excluded sites are included in the count, the mutation rate in the S1 gene increases up to 45.0%. Turquoise blue, yellow, blue, pink, and gray colors indicate reserved bases, substituted bases by point mutations, mutated bases with insertions, mutated bases based on insertion-and-deletion mutation with preserved base size, and mutated bases based on insertion-and-deletion mutations with changed base size, respectively.

**FIG 2:**
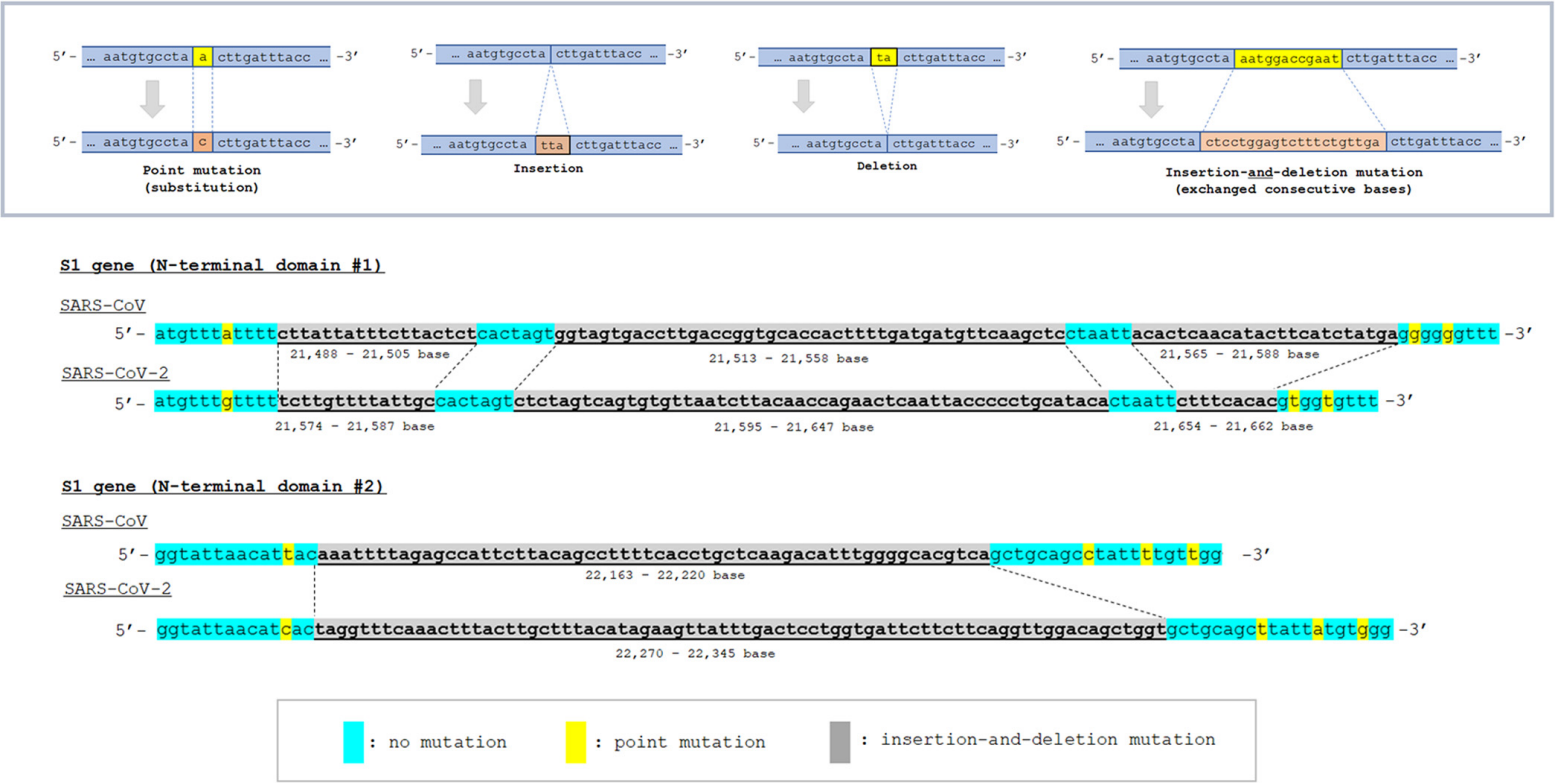
Examples of insertion-and-deletion mutations in the N-terminal domain of spike gene. In the present study, gene mutations were categorized into the following four general subtypes: point mutation, insertion, deletion, and insertion-and-deletion mutation. With an insertion-and-deletion mutation, consecutive bases were exchanged by totally different sequences with the same or different base size. Most of the observed insertion-and-deletion mutations in spike N-terminal domain involved ≥10 consecutive bases and resulted in changed base sizes.

**FIG 3:**
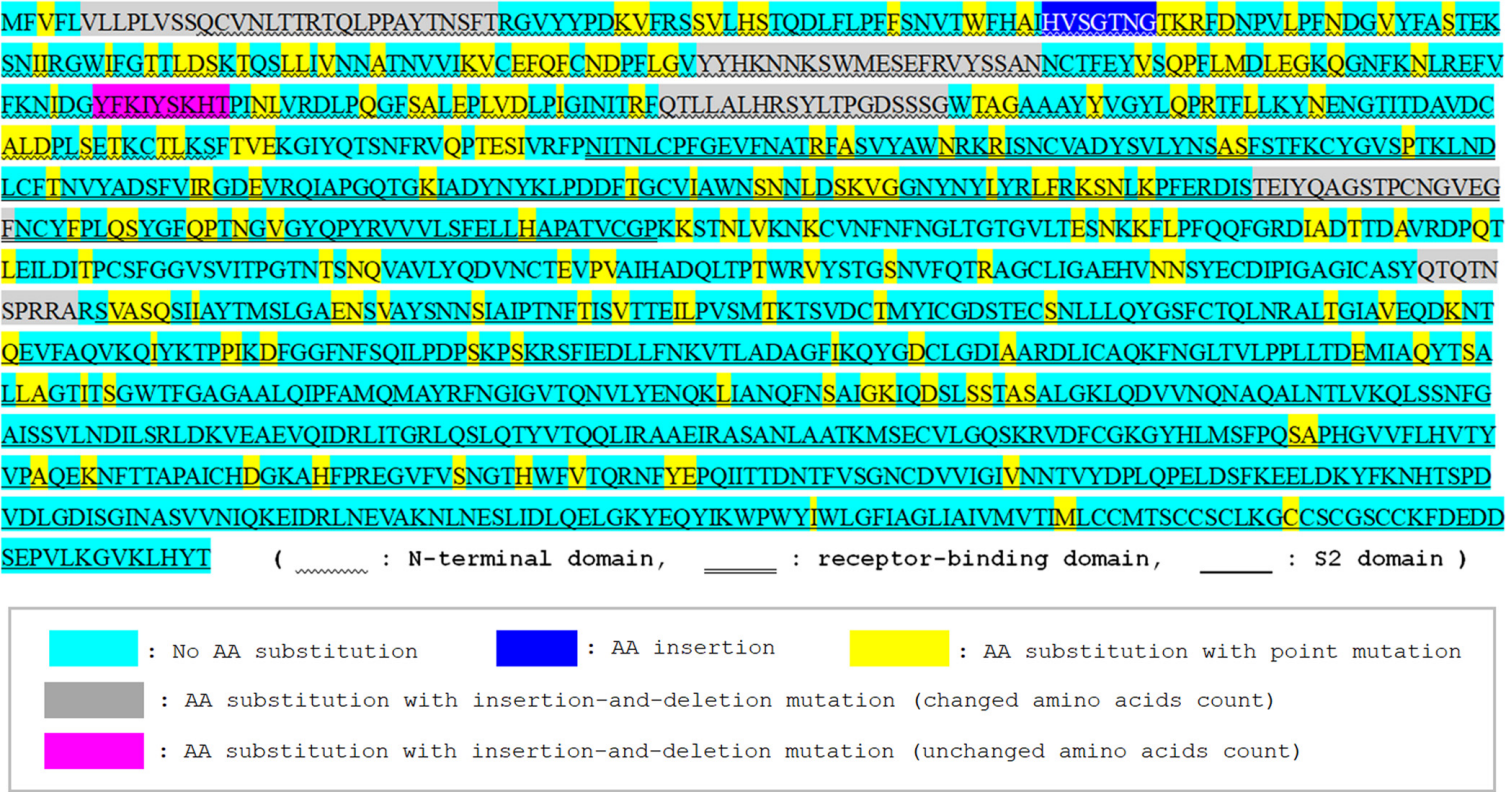
Amino acid substitution status in SARS-CoV-2 spike protein compared with SARS-CoV. The substitution status of amino acids (AA) in SARS-CoV-2 spike protein compared with those in SARS-CoV spike protein is shown. As similar to the base substitution status, amino acid substitutions were also concentrated in the S1 domain, especially in the N-terminal domain. The insertion-and-deletion mutations in S1 gene resulted in totally different amino acid sequences with the replacements of consecutive amino acids.

**FIG 4:**
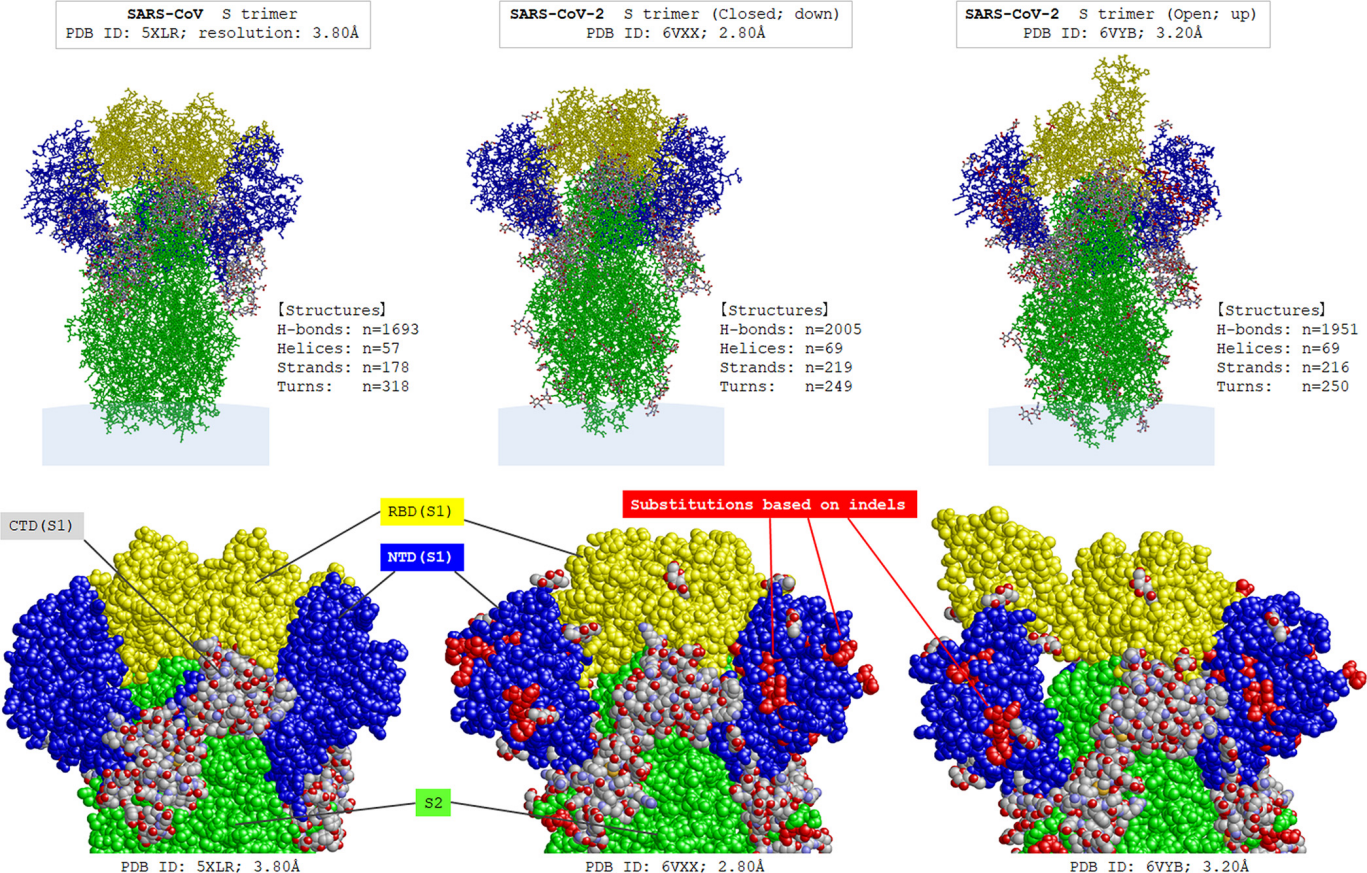
Molecular structures of the spike protein in SARS-CoV and SARS-CoV-2. Three-dimensional molecular structures of the S protein (closed state) in SARS-CoV and those in SARS-CoV-2 with closed and open states are shown. (Top) overall pictures of these proteins; (bottom) enlarged views of their S1 domains. The S1 NTD is shown in blue, receptor-binding domain (RBD) is in yellow, S2 domain is in green, and other subdomains including S1 CTD are in gray. The indel sites in SARS-CoV-2 S are shown as the consecutive amino acids colored in red. Conformational changes in SARS-CoV-2 spike NTD and RBD, compared with those in SARS-CoV, can be seen, and the RBD of SARS-CoV-2 are more centralized to the central pore than those of SARS-CoV. CTD, C-terminal domain; H-bonds, hydrogen bonds; indel: insertion and/or deletion; PDB, Protein Data Bank; S, spike.

The confirmed mutation sites and mutation types in the S gene between SARS-CoV and SARS-CoV-2 are shown in Fig. 1. All indels in the S gene of SARS-CoV-2 were also confirmed in RaTG13. A total of 11 indels were confirmed in the S gene; 9 (81.8%) were based on insertion-and-deletion mutations and 2 (18.2%) were based on insertions. As can be seen, most of the indels were concentrated in the N-terminal domain (NTD) of S1 gene. The actual insertion-and-deletion mutations in the NTDs of SARS-CoV S and SARS-CoV-2 S are shown in Fig. 2. As can be seen, most of the insertion-and-deletion mutations were accompanied by changed base sizes. The substitution status of amino acids in SARS-CoV-2 spike RBD, compared with the amino acids in SARS-CoV spike RBD, is shown in Fig. 3. The amino acids of the aforementioned insertion-and-deletion mutation sites were replaced by totally different amino acid sequences (gray color). In the 292 amino acids in the spike NTD of SARS-CoV-2, 152 (52.1%) were substituted (76 based on point mutations and 76 based on indels) from those in SARS-CoV spike NTD. In the 197 amino acids in the spike RBD, 53 (26.9%) were substituted (36 based on point mutation and 17 based on indels). In the 588 amino acids in S2 domain, 60 (10.2%; all based on point mutations) were substituted. The list of the types and position of amino acids that are suggested to be critical for binding to ACE2 receptors in the RBD of SARS-CoV and SARS-CoV-2 spike RBD is shown in Table 3. Among the 19 listed amino acids in SARS-CoV-2 spike RBD, 11 were substituted from those in SARS-CoV spike RBD. The binding surface of ACE2 is known to be negatively charged, and substitutions to positively charged amino acids are generally considered to stabilize the RBD-ACE2 binding (19, 20). Three-dimensional molecular structures of the S protein in SARS-CoV and those in SARS-CoV-2 with closed (“down”) and open (“up”) conformations are shown in Fig. 4. Conformational changes in SARS-CoV-2 spike NTD (blue color) and RBD (yellow color), compared with those in SARS-CoV, can be seen. The RBD of SARS-CoV-2 are more centralized to the central pore in the axial view than those of SARS-CoV. The indel sites in SARS-CoV-2 S protein are shown as the consecutive amino acids in the red color. With the insertion-and-deletion mutations, the number of amino acid in the spike NTD was increased from 279 to 292 amino acids, and this could have partially contributed to the conformational change in the spike NTD in SARS-CoV-2.

**TABLE 3:** Amino acids critical for binding to ACE2 receptors in the RBD of SARS-CoV and SARS-CoV-2 spike RBD^*a*^

2002–2003 SARS-CoV		Corresponding AA in SARS-CoV-2		AA substitution
SARS-CoV S (AA)	Characteristics of the R group	SARS-CoV-2 S (AA)	Characteristics of the R group	
V404	Val: nonpolar, aliphatic	K417	Lys: positively charged	Yes
T433	Thr: polar, uncharged	G446	Gly: nonpolar, aliphatic	Yes
Y436	Tyr: nonpolar, aromatic	Y449	Tyr: nonpolar, aromatic	No
K439	Lys: positively charged	L452	Leu: nonpolar, aliphatic	Yes
Y442	Tyr: nonpolar, aromatic	L455	Leu: nonpolar, aliphatic	Yes
Y440	Tyr: nonpolar, aromatic	Y453	Tyr: nonpolar, aromatic	No
P462	Pro: polar, uncharged	A475	Ala: nonpolar, aliphatic	Yes
P470	Pro: polar, uncharged	E484	Glu: negatively charged	Yes
L472	Leu: nonpolar, aliphatic	F486	Phe: nonpolar, aromatic	Yes
N473	Asn: polar, uncharged	N487	Asn: polar, uncharged	No
Y475	Tyr: nonpolar, aromatic	Y489	Tyr: nonpolar, aromatic	No
N479	Asn: polar, uncharged	Q493	Gln: polar, uncharged	Yes
D480	Asp: negatively charged	S494	Ser: polar, uncharged	Yes
G482	Gly: nonpolar, aliphatic	G496	Gly: nonpolar, aliphatic	No
Y484	Tyr: nonpolar, aromatic	Q498	Gln: polar, uncharged	Yes
T486	Thr: polar, uncharged	T500	Thr: polar, uncharged	No
T487	Thr: polar, uncharged	N501	Asn: polar, uncharged	Yes
G488	Gly: nonpolar, aliphatic	G502	Gly: nonpolar, aliphatic	No
Y491	Tyr: nonpolar, aromatic	Y505	Tyr: nonpolar, aromatic	No

a Amino acids type and position for those suggested to be critical for binding to ACE2 receptor in the amino acid sequences of SARS-CoV and SARS-CoV-2 spike RBD are shown. Among the listed 19 amino acids in SARS-CoV-2 spike RBD, 11 were substituted from those in SARS-CoV. Because the binding surface of ACE2 is known to be negatively charged, substitution to positively charged amino acids may contribute to stabilize the RBD-ACE2 interaction. AA, amino acid; ACE2, angiotensin-converting enzyme 2; R group, side chain; RBD, receptor-binding domain.

### Mutations in other structural genes of SARS-CoV-2.

**FIG 5:**
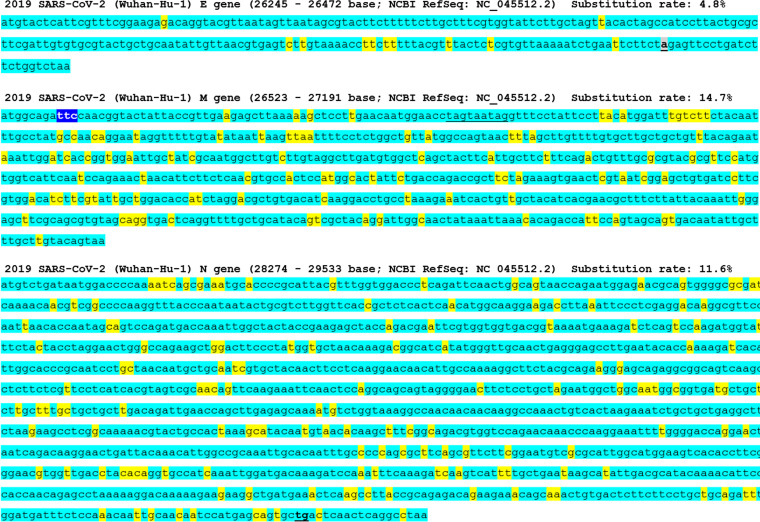
Sequences of structural proteins in SARS-CoV-2 and the mutations in SARS-CoV. The shown point substitution rates are for the sequences excluding the gray-colored insertion–deletion sites (sequences with preserved bases or point substitutions). Turquoise blue, yellow, and blue colors indicate reserved bases, bases substituted by point mutations, and bases with insertions, respectively.

Mutations in other structural genes, such as E, membrane (M), and N, are shown in Fig. 5. Compared to the mutation status in the S gene, the prevalence of point substitution and indels in these three genes was significantly lower between SARS-CoV and SARS-CoV-2. The overall mutation rates were 8.9%, 15.1%, and 11.6% for E, M, and N genes, respectively. The point substitution rates after excluding indel sites were 4.8%, 13.9%, and 11.6% for E, M, and N genes, respectively. The overall prevalence of mutations in each of the E, M, and N genes was significantly lower than that in the S gene (*P* < 0.0001, for all three genes, chi-square test).

### Mutations in nonstructural genes of SARS-CoV-2.

**FIG 6:**
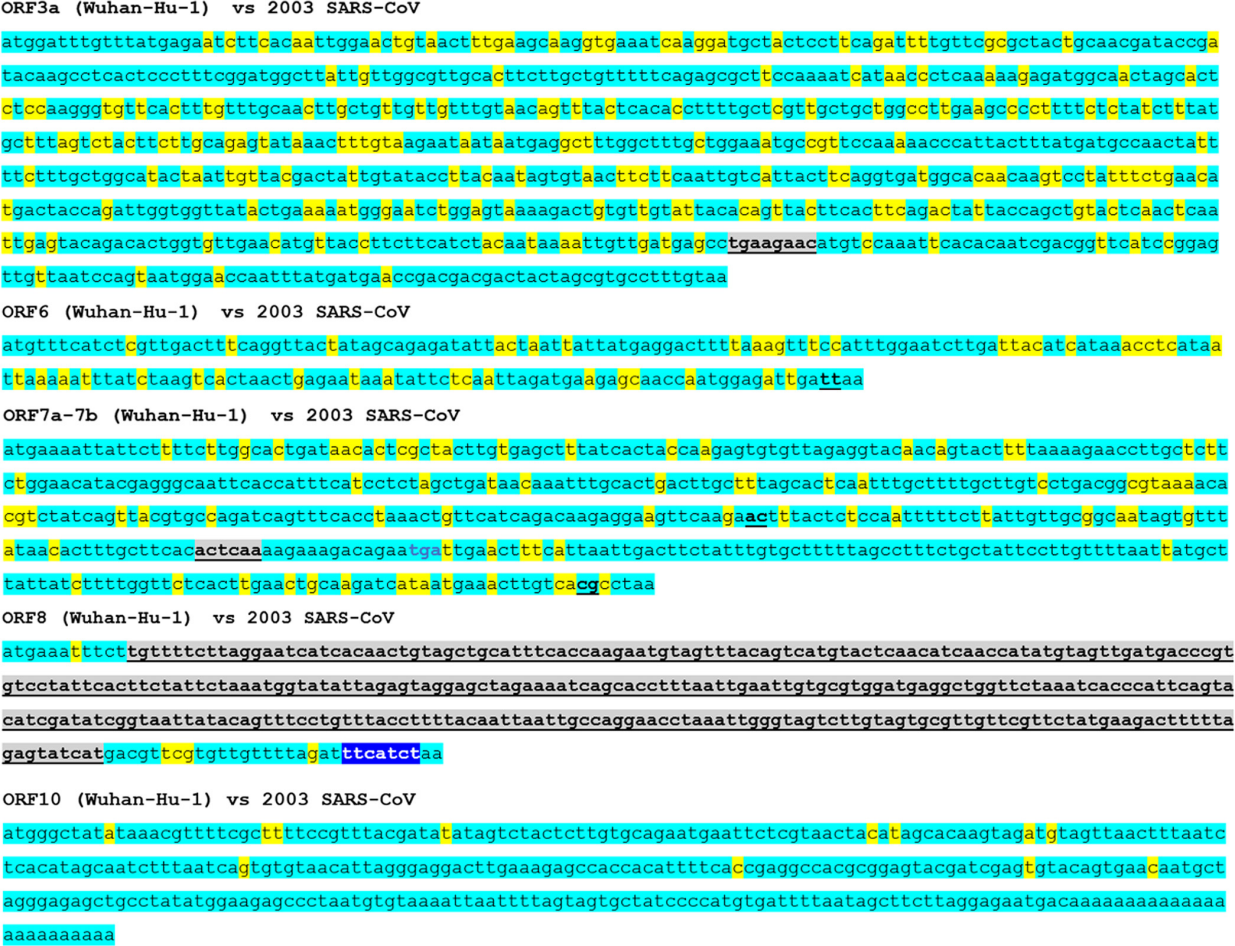
Sequences of ORF3a–ORF10 in SARS-CoV-2 and the mutations in SARS-CoV. The mutation rate in the ORF10 gene was significantly lower than that in other ORF family genes. Almost the complete ORF8 was substituted by a 333 consecutive base-long insertion-and-deletion mutation. Turquoise blue, yellow, blue, and gray colors indicate reserved bases, bases substituted by point mutations, bases with insertion, and mutated bases based on insertion-and-deletion mutations with changed base size, respectively. ORF, open reading frame.

Mutations in nonstructural genes between SARS-CoV and SARS-CoV-2 are shown in Fig. 6. Mutations in the ORF1ab genes were not shown in the figure, as the gene sizes were too large to show. Multiple long indels with 7–136 consecutive bases were concentrated in the Nsp3 gene of the ORF1a. Furthermore, almost the complete ORF8 gene was mutated via a 325 consecutive base long insertion–and-deletion mutation. This was the largest indel site in the entire genome of SARS-CoV-2.

### Mutations in the noncoding regions.

**FIG 7:**
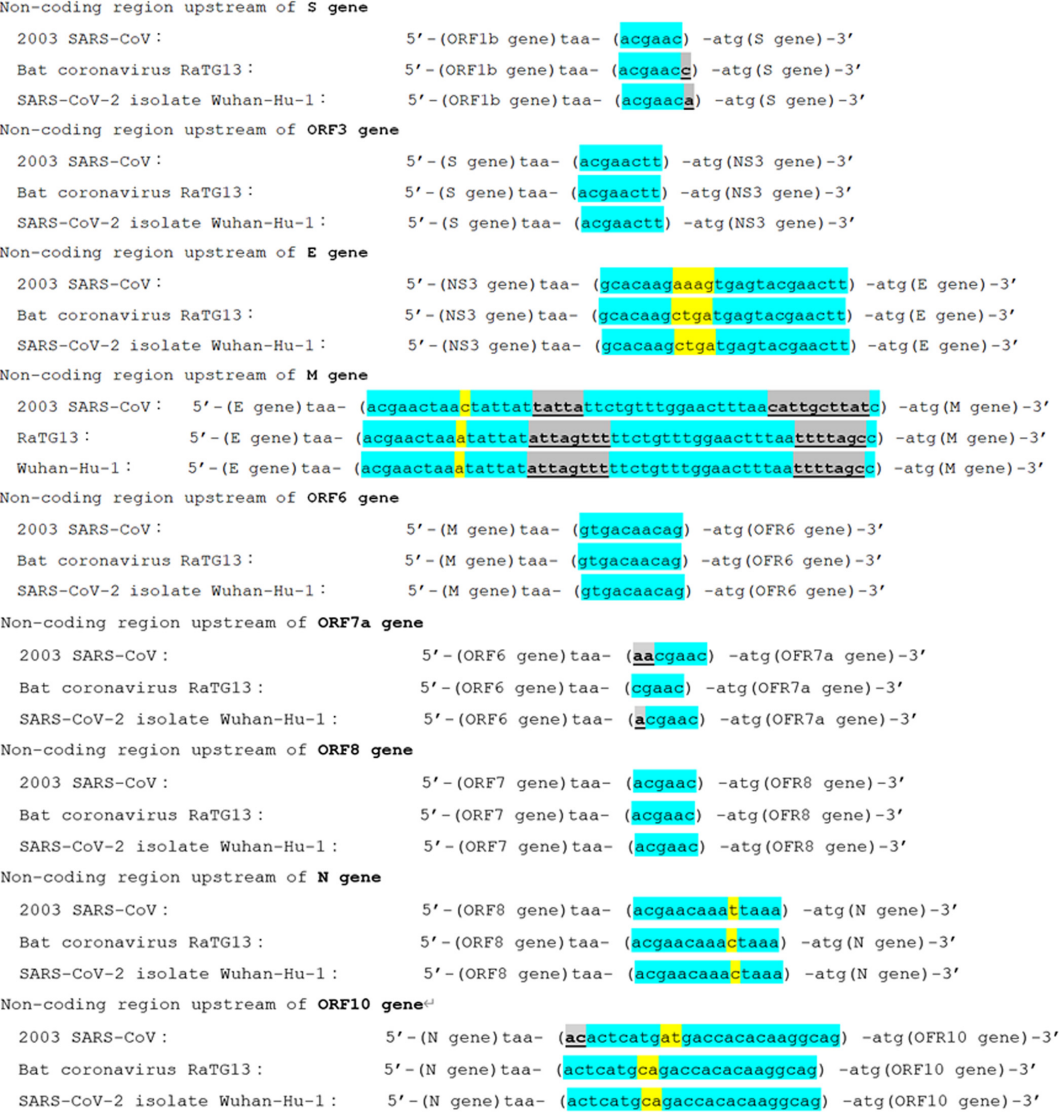
Evolution of mutations in noncoding regions in the three betacoronaviruses. Insertions or deletions occurred in four of the nine noncoding regions between coding genes, among which two of the three coincidentally occurred in the Kozak sequence-related positions (−3 to −1 positions from the start codon “aug”). One mutation upstream of the M gene realized the ideal Kozak motif of “gcc” in RaTG13 and SARS-CoV-2. Interestingly, noncoding regions upstream of the S gene and ORF7a gene contained different insertions or deletions at exactly the same position in RaTG13 and SARS-CoV-2. Turquoise blue, yellow, and gray colors indicate reserved bases, bases substituted by point mutations, and mutated bases based on insertion and/or deletion mutations with changed base size, respectively.

Mutations in the noncoding region upstream of each coding gene were studied and compared between the three betacoronaviruses. In the noncoding region upstream of the ORF1a gene in SARS-CoV-2 (that is, 1–265 bases), one short deletion site with the loss of three consecutive bases was confirmed. Other mutations in this region were all with point mutations. The point substitution rate in this noncoding region was 8.8%. The sequences in other noncoding regions in the three types of betacoronaviruses are shown in Fig. 7. Indels were confirmed in 5 of the 10 evaluated noncoding regions (upstream of ORF1a, S, M, ORF7a, and ORF10). Two indels occurred in the Kozak sequences (upstream of S and M genes), one of which gained the ideal Kozak consensus sequence motif of “-gcc-” in RaTG13 and SARS-CoV-2.

### Details of the abnormally long indels.

**FIG 8:**
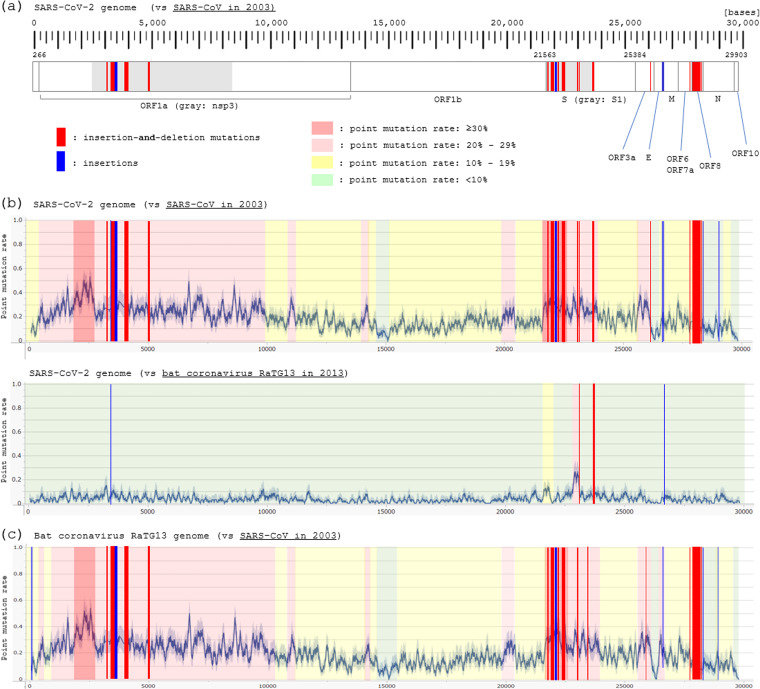
Distribution of insertion and/or deletion mutations on the genomes of SARS-CoV-2 and RaTG13. (a) Medium- to large-sized insertion and/or deletion mutations with approximately 10–300 consecutive base long were concentrated in the Nsp3 gene of the ORF1a, S1 domain of S gene, and ORF8 gene. (b) The line graphs show the simple moving average (±50 bases) for the point mutation rate across the genome of SARS-CoV-2, compared to the genome of SARS-CoV (top) or RaTG13 (bottom). The distribution of the indels (red and blue bars) was roughly matched to the distribution of the point mutation rate. (c) The line graph shows the simple moving average (±50 bases) for the point mutation rate across the genome of RaTG13, compared to that of SARS-CoV. The pattern of the point mutation rate and the distribution of indels roughly matched to those between SARS-CoV and SARS-CoV-2.

The indels with the involved base size of ≥10 consecutive bases in the whole genome, excluding the indels at the both ends of the genome (24 bases), are listed in Table 4. At least 16 indels with 10–325 consecutive bases were confirmed; 14 sites with insertion-and-deletion and 2 sites with insertions. Five sites were located in the Nsp3 gene of ORF1a gene, nine in the S1 gene, one in the S1/S2 boundary area, and another in the ORF8 gene. The possibility of inversion or duplication for the sequence of the insertion-and-deletion sites was checked, but it was not likely to explain the observed mutations. The distribution of the indel sites in the whole genome of SARS-CoV-2 is shown in Fig. 8a. Below the panel, line graphs of the moving average (±50 bases) for point substitution rates in each base position of SARS-CoV-2 genome are superimposed, when compared to SARS-CoV (Fig. 8b, top) or RaTG13 (Fig. 8b, bottom). The indels were concentrated in the gene regions with high point substitution rates and disproportionally distributed across the genome, suggesting the presence of evolutionary selection pressure behind the uneven distributions of the indels across the genome of SARS-CoV-2. Furthermore, the line graph of point mutation rate and the distribution of indels between the genomes of RaTG13 and SARS-CoV are shown in Fig. 8c, which were largely the same with those between the genomes of SARS-CoV-2 and SARS-CoV.

**TABLE 4:** Insertion and/or deletion mutations with ≥10 consecutive bases and with changed base size between SARS-CoV and SARS-CoV-2^*a*^

Virus and base position	Genome sequences with consecutive gene mutations
SARS-CoV-2 (3,064–3,070 b)	5′-aggtgat-3′ (Nsp3 gene of ORF1a)
SARS-CoV (3,051–3,060 b)	5′-cgatgcagag-3′
SARS-CoV-2 (3,216–3,351 b)	5′-gtcaacaaactgttggtcaacaagacggcagtgaggacaatcagacaactactattcaaacaattgttgaggttcaacctcaattagagatggaacttacaccagttgttcagactattgaagtgaatagttttag-3′ (Nsp3 gene of ORF1a)
SARS-CoV (3,206–3,346 b)	5′-ctgagcaatcagagattgagccagaaccagaacctacacctgaagaaccagttaatcagtttactggttatttaaaacttactgacaatgttgccattaaatgtgttgacatcgttaaggaggcacaaagtgctaatccta-3′
SARS-CoV-2 (3,365–3,441 b)	5′-cttactgacaatgtatacattaaaaatgcagacattgtggaagaagctaaaaaggtaaaaccaacagtggttgttaa-3′ (Nsp3 gene of ORF1a; insertion only with no corresponding sequence in SARS-CoV)
SARS-CoV-2 (3,826–3,955 b)	5′-ttcaagctttttggaaatgaagagtgaaaagcaagttgaacaaaagatcgctgagattcctaaagaggaagttaagccatttataactgaaagtaaaccttcagttgaacagagaaaacaagatgataag-3′ (Nsp3 gene of ORF1a)
SARS-CoV (3,744–3,867 b)	5′-catggattatcttgataacctgaagcctagagtggaagcacctaaacaagaggagccaccaaacacagaagattccaaaactgaggagaaatccgtcgtacagaagcctgtcgatgtgaagcca-3′
SARS-CoV-2 (4,880–4,894 b)	5′-agtaatcctaccaca-3′ (Nps3 gene of ORF1a)
SARS-CoV (4,792–4,809 b)	5′-ctggagagccccgtcgag-3′
SARS-CoV-2 (21,574–21,587 b)	5′-tcttgttttattgc-3′ (5′ terminal region, S1 gene)
SARS-CoV (21,488–21,505 b)	5′-cttattatttcttactct-3′
SARS-CoV-2 (21,595–21,647 b)	5′-ctctagtcagtgtgttaatcttacaaccagaactcaattaccccctgcataca-3′ (NTD, S1 gene)
SARS-CoV (21,513–21,558 b)	5′-ggtagtgaccttgaccggtgcaccacttttgatgatgttcaagctc-3′
SARS-CoV-2 (21,654–21,662 b)	5′-ctttcacac-3′ (NTD, S1 gene)
SARS-CoV (21,565–21,588 b)	5′-acactcaacatacttcatctatga-3′
SARS-CoV-2 (21,766–21,786 b)	5′-tacatgtctctgggaccaatgg-3′ (NTD, S1 gene; insertion only with no corresponding sequence in SARS-CoV)
SARS-CoV-2 (22,002–22,044 b)	5′-aaaacaacaaaagttggatggaaagtgagttcagagtttattc-3′(NTD, S1 gene)
SARS-CoV (21,922–21,952 b)	5′-tgggtacacagacacatactatgatattcga-3′
SARS-CoV-2 (22,159–22,186 b)	5′-ttattttaaaatatattctaagcacacg-3′(NTD, S1 gene)
SARS-CoV (22,067–22,094 b)	5′-gtttctctatgtttataagggctatcaa-3′
SARS-CoV-2 (22,270–22,345 b)	5′-taggtttcaaactttacttgctttacatagaagttatttgactcctggtgattcttcttcaggttggacagctggt-3′ (NTD, S1 gene)
SARS-CoV (22,163–22,220 b)	5′-aaattttagagccattcttacagccttttcacctgctcaagacatttggggcacgtca-3′
SARS-CoV-2 (22,974–22,991 b)	5′-aaatctatcaggccggta-3′ (RBD, S1 gene)
SARS-CoV (22,849–22,866 b)	5′-tgcctttctcccctgatg-3′
SARS-CoV-2 (23,004–23,014 b)	5′-atggtgttgaa-3′ (RBD, S1 gene)
SARS-CoV (22,879–22,886 b)	5′-ccccacct-3′
SARS-CoV-2 (23,590–23,630 b)	5′-tcagactaattctcctcggcgggcacgtagtgtagctagtc-3′ (S1/S2 furin cleavage site)
SARS-CoV (23,462–23,490 b)	5′-agtttctttattacgtagtactagccaaa-3′
SARS-CoV-2 (27,905–28,229 b)	5′-tgttttcttaggaatcatcacaactgtagctgcatttcaccaagaatgtagtttacagtcatgtactcaacatcaaccatatgtagttgatgacccgtgtcctattcacttctattctaaatggtatattagagtaggagctagaaaatcagcacctttaattgaattgtgcgtggatgaggctggttctaaatcacccattcagtacatcgatatcggtaattatacagtttcctgtttaccttttacaattaattgccaggaacctaaattgggtagtcttgtagtgcgttgttcgttctatgaagactttttagagtatcat-3′ (ORF8 gene)
SARS-CoV (27,775–28,067 b)	5′-cattgttttgacttgtatttctctatgcagttgcatatgcactgtagtacagcgctgtgcatctaataaacctcatgtgcttgaagatccttgtaaggtacaacactaggggtaatacttatagcactgcttggctttgtgctctaggaaaggttttaccttttcatagatggcacactatggttcaaacatgcacacctaatgttactatcaactgtcaagatccagctggtggtgcgcttatagctaggtgttggtaccttcatgaaggtcaccaaactgctgcatttaga-3′

a Mutations listed in the table are those with insertion and/or deletion mutations involving ≥10 consecutive bases. Most of these mutations were accompanied by changed base sizes. These mutations in ORF1 were concentrated in the Nsp3 gene region. In the 13 listed long insertion and/or deletion mutations in the S gene, 9 occurred in the N-terminal domain (NTD), 3 occurred in the receptor-binding domain, and 1 occurred in the S1/S2 boundary region that produced the furin cleavage site in SARS-CoV-2. Repeating sequences that may promote a large length of insertions were not apparent in the listed mutation sites. Nsp3, nonstructural protein 3; b, base.

## DISCUSSION

Generally, the incidence of replication error during RNA virus proliferation increases up to 10^−5^ to 10^−4^ errors per base replication (21, 22). This is because most virus genome-encoded RNA-dependent RNA polymerases cannot repair the errors themselves. The SARS-CoV-2 genome contains genes encoding nonstructural protein Nsp12 and Nsp14, which collaboratively function to repair replication errors; thus, the estimated mutation rate of SARS-CoV-2 is currently approximately 10^−6^ to 10^−5^ errors per base replication (23–25). The current estimate of mutation rate in SARS-CoV-2 after adjusting for the effects of evolutionary selection was approximately 10^−3^ mutations per site per year (24, 26, 27), which was comparable to that in SARS-CoV (28). Based on this estimation, the observed mutation rate in this study across the coding regions of structural genes and noncoding regions between SARS-CoV and SARS-CoV-2 or RaTG13 seems to be significantly high as a natural evolutionary process, if we simply regard that SARS-CoV is a progenitor of SARS-CoV-2. This study revealed that at least 17 of the 35 indel sites were based on insertion-and-deletion mutations and were concentrated in the NTD of the S1, Nsp3 gene of the ORF1a, and ORF8 genes. The comparison of the three-dimensional molecular structures between the NTD of the S1 gene in SARS-CoV and that in SARS-CoV-2 suggested that the conformation of the domain was largely changed by the indels, possibly further affecting the conformation of the adjacent RBD. Although the exact molecular effect of this conformational change to the binding capacity of the RBD is uncertain, the conformational change could have affected the binding capacity of S protein against ACE2 in SARS-CoV-2, compared to that in SARS-CoV (29, 30). The strength of RBD-ACE2 binding, evaluated by the dissociation constant, showed discrepancies between different experimental approaches (surface plasmon resonance versus biolayer interferometry binding) (31). Another recent study utilizing molecular dynamic simulation suggested that the surface of SARS-CoV-2 spike RBD is more positively charged and more attractive to the negatively charged surface of ACE2 than that of SARS-CoV, but the simulated total electrostatic forces between spike RBD and ACE2 were stronger in SARS-CoV than in SARS-CoV-2 (32). In spite of these inconsistent experimental results, the binding of RBD to ACE2 is still thought to be easier in SARS-CoV-2 than in SARS-CoV, as the sequence change in the hinge between RBD and other S domains could have made the RBD more flexible and easier to open for binding ACE2 (32). Furthermore, if the two viruses were derived from reservoir animals like bats or pangolins, these conformational changes in RaTG13 or SARS-CoV-2 S protein may have further granted immune evasion to the viruses.

This study also revealed that almost the complete ORF8 gene was substituted, based on an approximately 325 consecutive base-long insertion-and-deletion mutation. Although the genome sequences of ORF8 differ significantly between different coronaviruses and exact function of ORF8 remain unknown, the persistence of ORF8 in different lineages is proposed to suggest that it may play some unknown roles in SARS-CoV-2 replication (33). Meanwhile, the finding of this study that almost all of the genome sequence of ORF8 in SARS-CoV-2 was mutated from those in SARS-CoV may support that ORF8 is possibly dispensable for SARS-CoV-2 survival. In general, indels are less frequent than point substitutions in the natural environment, and when indels occur, most of the mutations are with relatively short length of consecutive bases. One of the reasons for this is that an indel would result in a frameshift in most cases, which usually causes a fatal change to the subsequent amino acid sequence, unless the change in base size is a multiple of three. Certainly, an up to 10,000 base-long indel can be observed in some eukaryotes as a natural evolutionary process. The presence of multiple indels in the genome can be a rationale for determining species and building phylogenetic trees (34–36). Some indels may have affected the evolvability of the involved species (37). The exact mechanisms of the occurrence of long indels in the natural environments remains uncertain, but the recently introduced genetic engineering technique using clustered regularly interspaced short palindromic repeats (CRISPR) and CRISPR-associated proteins (Cas) may partially explain the phenomenon (38, 39). The CRISPR technology was originally developed from the bacterial CRISPR-Cas9 antiviral immune system, which had already existed in almost all archaea and many bacteria in the natural environment (40–43). The CRISPR-Cas system is useful in RNA and DNA editing that realized relatively precise genome modifications, including an induction of indel to the DNA sequences (44, 45). The CRISPR toolkit is further expected to shorten the time for developing an effective live attenuated vaccine for some viruses or realizing viral load reduction after viral infections (45, 46). However, whether the CRISPR-Cas13 system (which targets RNA and not DNA) can realize the long insertion-and-deletion mutations in RNA molecules that were observed in the present study remains unknown. Conceivable hypotheses to explain the observed concentrated indels in the spike NTD of RaTG13 and SARS-CoV-2 include a natural evolutionary process. Generally, most indels are more deleterious than point substitution and vulnerable to evolutionary selection pressure (47). If indels occur in the coding regions with less selective tolerance, such as the structural core proteins and other proteins that are essential for survival, most of such deleterious mutations would surely be selected out (48). This may explain why indels were so concentrated in the NTD of the S1 gene in RaTG13 and SARS-CoV-2.

Another notable finding of this study was that insertion and/or deletion was confirmed in five of the 10 evaluated noncoding regions upstream of coding regions, 2 of which occurred in the Kozak sequences just before the start codon. Mutations upstream of S gene changed the Kozak sequence in −3 to −1 positions from “-aac-” (SARS-CoV) to “-acc-” (RaTG13) and that of M gene from “-atc-” (SARS-CoV) to “-gcc-” (RaTG13). These sequences are exactly the same with the “strong” Kozak consensus sequence motifs known to promote protein translation initiation by facilitating the assembly and translation start of the ribosome. Kozak sequences significantly regulate translation efficiency in eukaryotes (49–51). As the viruses depend on the host cells for their translation machinery, it is reasonable to expect that the translation efficiency of SARS-CoV-2 is also significantly influenced by the Kozak sequences upstream of coding regions. Furthermore, two insertion and/or deletion mutations occurred at the same position between RaTG13 and Wuhan-Hu-1 (noncoding regions upstream of the S gene and ORF7a gene). Whether such coincidence can occur in the natural environment remains uncertain, but this finding may be a clue to consider the possible evolutionary mechanism of RaTG13 and SARS-CoV-2.

This study has some limitations. First, the exact numbers of the inducted insertion-and-deletion mutation in the S, Nsp3, and ORF8 genes may not be definite, as some of the long insertion-and-deletion mutations may comprise several different mutations in the same positions. Furthermore, the exact roles of Nsp3 and ORF8 have not been fully elucidated. As a result, the exact biological and physiological significance of the observed concentrated insertion-and-deletion mutations in these genes in relation to SARS-CoV-2 development remains uncertain. As another limitation, this study only evaluated the genome sequence of three betacoronaviruses. Recently, genome sequences of the betacoronavirus sampled from *Rhinolophus shameli* bats in Cambodia in 2010 (RshSTT182 and RshSTT200) were reported to share 92.6% identity with SARS-CoV-2, implying that the progenitors of SARS-CoV-2 could have wider geographic distribution than previously expected, extending from Southeast Asia to southern China (52). To further elucidate the evolutionary process of SARS-CoV-2, genome sequence analysis with further betacoronaviruses that may lie between SARS-CoV and SARS-CoV-2 in the phylogenetic tree, sampled from diverse rhinolophid bats species in different countries, will be essential (53). Lastly, the coronavirus gene expression cannot be judged simply by the gene sequence or translation efficiency. There are many other viral factors that may affect gene expression levels, such as the functions of proteases coded in the viral RNA, viral subgenomic RNA amount, and RNA secondary structures (54–57). These unevaluated factors should also be compared between SARS-CoV and RaTG13 or SARS-CoV-2 to conclude the effect of the observed gene mutations on the changes in gene expression levels between the three betacoronaviruses.

In conclusion, mutations between SARS-CoV and SARS-CoV-2 were unevenly distributed across the virus genome. In the S1 gene, at least nine indels with greater or equal to seven consecutive bases long, including eight indels based on insertion-and-deletion mutations, were concentrated. Replacement of relatively long consecutive bases with insertion-and-deletion mutations were also confirmed in ORF1a (Nsp3) and ORF8 genes. The uneven distribution of medium- to large-sized indels based on insertion-and-deletion mutations in the SARS-CoV-2 genome may provide further insights into the evolutionary process of the betacoronavirus in the last decades. Future studies comparing SARS-CoV-2 genome with viral genomes of various betacoronaviruses, collected from wide range of animals, and evaluating the accumulation process of the observed insertion-and-deletion mutations in SARS-CoV-2 genome may offer us further insights into the process of SARS-CoV-2 development.

## MATERIALS AND METHODS

### Genome sequence references.

The genome sequence of the SARS-CoV-2 isolate Wuhan-Hu-1, the National Institute of Health (NIH) genetic sequence database (GenBank) was referenced (reference sequence: NC_045512.2) (6, 58). The GenBank database for bat coronavirus RaTG13 was also referenced (reference: MN996532.2) (8, 59). The SARS-CoV genome sequence was obtained from a previous report by the Centers for Disease Control and Prevention and GenBank database (reference: AY345986.1) (60–62).

### Evaluated genome regions.

This study investigated all genome sequences and compared the sequences of SARS-CoV isolate CUHK-AG01, RaTG13, and SARS-CoV-2 isolate Wuhan-Hu-1. The evaluated specific genes were as follows: ORF1ab (266–21,555 bases in Wuhan-Hu-1), S (S1 and S2 subunits; 21,563–25,384 bases), ORF3a (25,393–26,220 bases), E (26,245–26,472 bases), M (26,523–27,191 bases), ORF6 (27,202–27,387 bases), ORF7a (27,394–27,759 bases), ORF8 (27,894–28,259 bases), N (28,274–29,533 bases), and ORF10 (29,558–29,674 bases). In addition to these coding regions, the sequences of the noncoding region upstream of ORF1a, S, ORF3, E, M, ORF6, ORF7a, ORF8, N, and ORF10 were further compared.

### Types and rate of mutations.

The mutations in the sequences of SARS betacoronaviruses were classified into indels and point mutations. Indels were further classified into insertion, deletion, and insertion-and-deletion mutations. Most of the insertion-and-deletion mutations resulted in changed base sizes, but some of them did not. Other popular mutations, such as duplication, inversion, or repeat expansion, were not apparent in the genome sequences evaluated in this study. Mutations in greater than or equal to seven consecutive bases in the coding regions were considered not to be a coincidental repetition of single base substitution, based on the knowledge that approximately 80% genome sequences were identical between SARS-CoV and SARS-CoV-2. Therefore, the probability of coincidental repetition of seven consecutive bases based on coincidental repetitive single base substitution is (1/5)^7^ = 1/78,125, which is sufficiently low for the size of each evaluated coding gene in this study. Substitutions in less than or equal to six consecutive bases with preserved base size were regarded as coincidental repetition of point mutations, as the probability is not too low to reject the hypothesis of coincidental repetition.

### Point substitution rate by genes.

In each of the genes, point substitution rate in different gene positions were evaluated. In the genes with concentrated indels inside (i.e., ORF1ab and S genes), line graphs for the point substitution rates by the positions across the genes were depicted. In these two genes, the line graphs of the point substitution rate were obtained by calculating the simple moving average of the 50 bases before and after a specific base (i.e., ±50 bases for each base). Then, the simple moving average of point mutation rate at the base position of *k* (*SMA_k_*) can be described as below by using the point mutation status (0 or 1) at the base position of *k* (*M_k_*):

$$\mathrm{SM}A_{k}=\;\frac{1}{100}\overset{49}{\underset{i=-50}{\sum }}M_{k+i}$$

The levels of the moving average of point substitution rates were categorized into the following four groups: very high (≥30%), high (20% to 29%), moderate (10% to 19%), and low (<10%).

### Three-dimensional molecular structures of the S protein.

The three-dimensional molecular structures were built and compared between the betacoronaviruses by using RasMol Software (http://www.openrasmol.org/). The structures for the S proteins of SARS-CoV and SARS-CoV-2 were obtained from RCSB Protein Data Bank with the Protein Data Bank file format (https://www.rcsb.org/) (16, 63). General appearance of the secondary, tertially, and quaternary structures of the S protein, in relation to the sites of the observed insertion-and-deletion mutations, was then visually evaluated and compared between that in SARS-CoV and in SARS-CoV-2.

### Statistical analyses.

The frequency of base substitution in a specific gene between different types of coronaviruses was compared by the chi-square test, using R Statistical Software (version 4.0.5; R Foundation, Vienna, Austria). Statistical significance was set at *P* < 0.05.

### Data availability.

This study does not use original sequencing data. All used sequencing data can be obtained from the NIH GenBank homepage. Genome sequence of the SARS-CoV-2 (Wuhan-Hu-1) is available at https://www.ncbi.nlm.nih.gov/nuccore/1798174254. Genome sequence of the bat coronavirus RaTG13 is available at https://www.ncbi.nlm.nih.gov/nuccore/MN996532. Genome sequence of the SARS-CoV (CUHK-AG01) is available at https://www.ncbi.nlm.nih.gov/nuccore/AY345986.
